# The transcription factor GmNAC018 confers salt tolerance in soybean

**DOI:** 10.3389/fpls.2025.1728235

**Published:** 2026-01-19

**Authors:** Lianjia Zhao, Xingang Li, Peiguo Wang, Weiwei Hao, Gan Li, Chunyan Li, Daolong Liao, Ke Wen

**Affiliations:** 1 Crop Research Institute of Xinjiang Uygur Autonomous Region Academy of Agricultural Sciences, Urumqi, Xinjiang, China; 2 Sanya Research Institute, Hainan Academy of Agricultural Sciences, Sanya, Hainan, China; 3 Key Laboratory of Vegetable Biology of Hainan Province, Vegetable Research Institute of Hainan Academy of Agricultural Sciences, Haikou, Hainan, China; 4 The Guangdong Subcenter of the National Center for Soybean Improvement, College of Agriculture, South China Agricultural University, Guangzhou, Guangdong, China; 5 Sanya National Nanfan Research Institute, Chinese Academy of Agricultural Sciences, Sanya, Hainan, China; 6 Institute of Agricultural Quality Standards and Testing Technology, Xinjiang Academy of Agricultural Sciences, Urumqi, Xinjiang, China; 7 Xinjiang Xinlianxin Chemical Industry Co., Ltd, Changji, Xinjiang, China; 8 Bureau of Agriculture and Rural Affairs of Chabucha’er County, Lli, Xinjiang, China

**Keywords:** GmNAC018, molecular mechanism, salt stress, soybean, transcription factor

## Abstract

Soybean (*Glycine max*) plays a vital role in global food security and nutrition but faces increasing threats from soil salinization, an expanding environmental issue that affects approximately 1.38 billion hectares of land worldwide. Plants possess a diverse array of genes that facilitate adaptation to salt stress. Transcription factors are central regulators in mediating this response. They coordinate the physiological process that enables plants to adapt to high-salt environments by regulating the expression of downstream genes. The present study examines the function of the *GmNAC018* gene in improving salt tolerance in soybean. We generated transgenic soybean plants with overexpression and observed improved germination, higher chlorophyll content, increased biomass, and lower sodium ion accumulation under salt stress compared to wild-type plants. The overexpression of *GmNAC018* resulted in increased expression of key salt-tolerance genes, demonstrating its involvement in activation of the salt stress response pathway. Our results also suggest that *GmNAC018* may be a key gene in regulating salt tolerance in soybean, providing theoretical molecular support for understanding the salt tolerance regulation mechanism in plants.

## 1 Introduction

According to statistics, the global area of land affected by salinization at varying degrees has reached approximately 1.38 billion hectares, accounting for 10.7% of the total land area worldwide (Basak et al., 2022; Singh, 2022; Negacz et al., 2022). Furthermore, climate change and inadequate land management practices have placed an additional 1 billion hectares of land at risk of salinization, which threatens global agricultural productivity (Okur and Örçen, 2020; Teh and Koh, 2016). Soybean (*Glycine max*) is a globally significant crop cultivated for both oil and grain production. It supplies approximately 70% of plant-derived proteins and 30% of vegetable oils consumed by humans, establishing its role as a key crop for food security and balanced dietary nutrition (Kim et al., 2021; Dukariya et al., 2020). Previous research has established that soybean exhibits moderate tolerance to salt stress (Parida and Das, 2005). In the short term, salt stress rapidly induces osmotic stress in plants, leading to a decrease in root water absorption potential and reduced water uptake efficiency, which results in physiological water deficit in plants (Xiong and Zhu, 2002; Rasool et al., 2012). In the long term, salt stress disrupts plant ion homeostasis, particularly through the excessive accumulation of sodium ions (Na^+^) in cells, which triggers toxic effects (Arif et al., 2020; Mushtaq et al., 2020). This process disrupts the absorption and translocation of essential mineral elements, including potassium ions (K^+^), and leads to substantial production of reactive oxygen species (ROS). Ultimately, this process results in cellular oxidative damage, reduced plant growth, and substantial decreases in both soybean yield and quality.

Recent studies have made significant advances in elucidating the molecular mechanisms underlying soybean salt stress tolerance. Recent research has concentrated on key areas such as salt stress signal perception, precise regulation of ion homeostasis, and activation of antioxidant defense pathways. These advances form a foundational framework for elucidating the molecular genetic mechanisms underlying soybean salt tolerance (Phang et al., 2008). According to previous reports, *GmNTL1* can sense the secondary signal of salt stress through oxidative modification in signal transduction (Zhang et al., 2024). In terms of ion homeostasis regulation, the plasma membrane Na^+^/H^+^ antiporter *GmSOS1* (Salt Overly Sensitive 1 (SOS1) Na^+^/H^+^ antiporter) helps maintain cellular ion balance by mediating sodium efflux, while genes such as *GmSALT3* and *GmCHX1* reduce ionic toxicity by limiting Na^+^ and Cl^-^ accumulation in shoots (Zhang et al., 2022; Guan et al., 2014; Qi et al., 2014; Do et al., 2016). Furthermore, the GmCBL9–GmCIPK6 complex enhances K^+^ uptake and translocation through activation of the *GmAKT1* channel, thereby improving the Na^+^/K^+^ ratio under salt stress (Feng et al., 2025). In terms of antioxidant defense activation, the NADP^+^-dependent malate dehydrogenase *GmMDH2* and glutathione S-transferase *GmGSTU23* enhance the activities of antioxidant enzymes such as superoxide dismutase (SOD) and catalase (CAT) by utilizing the intracellular NADPH and reduced glutathione pool, effectively reducing ROS accumulation (Zhou et al., 2024; Li et al., 2023). The LRR-RLK gene *GmHSL1b* enhances salt tolerance by modulating root meristem activity and increasing reactive oxygen species scavenging efficiency. These mechanisms maintain reactive oxygen species homeostasis and promote adaptive root development (Ma et al., 2025).

Transcription factors (TFs) are a class of key regulatory proteins that can specifically bind to specific cis-acting elements (e.g., core motifs, response elements) in the promoter region of target genes through their conserved DNA-binding Domains (DBDs). This binding further regulates the transcriptional activation or repression of downstream target genes, enabling TFs to play a core regulatory role in the gene expression network governing plant growth and development, as well as stress responses (Yadavab et al., 2016).​ The NAC family, named after the representative members NAM, ATAF1/2, and CUC2, constitutes one of the most abundant and multifunctional transcription factor families in higher plants. The number of NAC family members has been identified in various species, with notable variation observed among wild emmer wheat (*Triticum dicoccoides* L.), maize (*Zea mays*), sorghum (*Sorghum bicolor*), and rice (*Oryza sativa*) (Rui et al., 2023; Shiriga et al., 2014; Sanjari et al., 2019; Nuruzzaman et al., 2010). In soybean (*G. max*), 139 NAC family transcription factors have been identified via whole-genome sequence analysis (Hussain et al., 2017). These members are involved in regulating soybean growth and development, as well as defense responses to biotic and abiotic stresses such as salt, drought, and low temperature, forming a complex functional regulatory network (Hussain et al., 2017; Miransari, 2015; Miransari, 2016).​ For example, *GmNAC039* activates the transcription of *GmCYP* (cytochrome P450 (CYP) enzyme family), thereby promoting nodule senescence (Yu et al., 2023; Doll, 2023). *GmNAC181* can also reduce the damage of salt stress to the symbiotic nitrogen fixation system by maintaining root morphogenesis and nodule structure stability under salt stress (Wang et al., 2022). In contrast, *GmNAC115* and *GmNAC3* enhance the tolerance to drought stress by regulating related stress response genes and antioxidant activity (Wan et al., 2025; Amin et al., 2024). *GmNAC1* activates the expression of *GmDR1*, which enhances soybean resistance to *Phytophthora sojae.* (Yu et al., 2022). While the functions of some soybean NAC family transcription factors have been relatively clearly characterized, the specific molecular mechanisms underlying their involvement in soybean salt stress tolerance remain to be further elaborated.

In the present study, a salt-responsive NAC transcription factor named *GmNAC018* was isolated from soybean, and its response to salt stress was detected by quantitative real-time PCR (qRT-PCR). To elucidate the function of *GmNAC018*, the phenotype of overexpressed transgenic lines from soybean was analyzed. In addition, under salt stress, the expression levels of six key salt-tolerance related genes in overexpression (OE) lines were significantly higher than those in wild type (WT) plants. This discovery suggests that the overexpression of *GmNAC018* may enhance the salt tolerance of soybean by activating these genes. The results offer theoretical support for elucidating the role of NAC transcription factors in the molecular mechanisms underlying salt tolerance in plants.

## 2 Materials and methods

### 2.1 Plant material and growth conditions

This study used the Huachun 6 soybean variety from South China Agricultural University to analyze the expression pattern and develop transgenic lines of *GmNAC018* (*Glyma.04G208300*). Seeds were germinated in vermiculite. Uniform seedlings were selected and transferred to a hydroponic system containing modified Hoagland nutrient solution at pH 6.8. The system was maintained at 28°C during the day and 25°C at night, with a 14-hour light and 10-hour dark cycle. The nutrient solution was replaced daily (Li et al., 2023). To assess tissue-specific expression of *GmNAC018*, roots, stems, and leaves were collected at the V_1_ growth stage (Cai et al., 2020). For time-course analysis under salt stress, plants were treated with 150 mM NaCl, and roots and leaves were sampled at 0, 1, 3, 6, 12, and 24 hours post-treatment. All samples were immediately frozen in liquid nitrogen and stored at -80°C for further analysis.

### 2.2 Expression patterns analysis

Total RNA was extracted with RNA-easy Isolation Reagent (Takara, Shiga, Japan), and cDNA was synthesized using the PrimeScript RT kit (Vazyme, Nanjing, China). qRT-PCR was performed on a CFX96™ Touch Real-Time PCR System (Bio-Rad, Hercules, CA, USA) with Taq Pro Universal SYBR qPCR Master Mix (Vazyme, Nanjing, China). Three biological replicates were included for each sample. The reaction conditions were as follows: pre-denaturation at 95°C for 30 s, denaturation at 95°C for 5 s, and annealing at 60°C for 30 s, followed by 40 cycles. All reactions were conducted in triplicate. Relative gene expression levels were determined using the 2^-△△ct^ method with GmACTIN6 (GeneBank Accession: AAK285830.1) as an internal control. Primers were designed using the NCBI Primer tool (https://www.ncbi.nlm.nih.gov/tools/primer-blast/, accessed on 13 May 2024) and were synthesized by Sangong Biotech Co., Ltd (Guangzhou, China) (**Supplementary Table S2**).

### 2.3 Cloning and bioinformatics analysis

The protein sequences of the NAC family were downloaded from the Phytozome 13.0 database (https://phytozome-next.jgi.doe.gov/, accessed on 26 January 2025). To explore the evolutionary relationships among NAC genes in different plant species, an evolutionary tree for the NAC protein family were constructed using the neighbor-joining (NJ) method with a bootstrap value of 1000, implemented in MEGA 11 software. Physiochemical parameters of GmNAC018 protein, including polypeptide length, molecular weight, isoelectric point, etc., were analyzed using ExPASy (http://web.expasy.org/protparam/, accessed on 27 January 2025).

### 2.4 Transgenic lines generation

The coding sequence (CDS) of *GmNAC018* was cloned from the soybean cultivar Huachun 6 and inserted into the pTF101 vector, which contains a modified CaMV 35S promoter and the BAR resistance gene as a selective marker for transgenic lines. The 35S:NAC018 construct was introduced into Agrobacterium tumefaciens strain EHA105 (Gene Pulser Xcell™ Electroporation Systems, Hercules, CA, USA) and subsequently used to transform soybean variety Huachun 6 following the protocol described by Chen et al. (2018). The soybean overexpression vector used in this study contains the glufosinate-resistant bar gene. Therefore, positive seedling identification was conducted via the smearing method using glufosinate-ammonium (Liberty^®^, Bayer, Leverkusen, Germany). A 250 mg/L Basta solution was smeared on half of a leaf of the transgenic plants. After approximately 4 days, leaf growth was observed. If half of the leaf smeared with Basta turned yellow and withered, the seedling was considered a false positive. If the growth status of the left and right sides of the leaf was consistent, the seedling was identified as a positive seedling. Subsequently, shoot apical meristems were sampled from the OE lines and the WT Huachun 6 at the V_3_ stage of soybean. RNA extraction, reverse transcription, and qRT-PCR reactions were performed. The expression level of *GmNAC018* in Huachun 6 was set as the control (with a value of 1), and *GmACTIN6* (GenBank accession number: AAK285830.1) was used as the reference gene. Each sample was analyzed in three biological replicates. The relative expression level was calculated using the 2^-ΔΔCt^ method (Cai et al., 2020). Afterwards, the three lines showing the highest relative expression levels of *GmNAC018* were then selected for further phenotypic analysis.

### 2.5 Subcellular localization

The subcellular localization of the GmNAC018 protein was predicted using the online tool Plant-mPLoc (http://www.csbio.sjtu.edu.cn/bioinf/plant-multi/, visited on January 27th, 2025). Subsequently, the GmNAC018 (lacking termination codon) gene was inserted into the 5’-terminus of the GFP gene in the pCMBIA1302-GFP vector to form a recombinant plasmid. It was transformed into *Agrobacterium tumefaciens* GV3101 by the freeze-thaw method and then injected into tobacco (*Nicotiana benthamiana*) leaves (Chen et al., 2022). Moreover, confirmed by molecular identification (**Supplementary Table S2**). Following a 48-hour dark incubation, transient expression of the fusion protein was examined using a laser confocal microscope (Lecia TCS SP8 STED, Lecia, Solms, Germany). The nucleus is labeled with DAPI dihydrochloride (Aladdin, Shanghai, China).

### 2.6 Phenotypes analysis of the transgenic line

We evaluated the germination rates of three *GmNAC018* OE lines and the WT under salt stress at 0, 100, and 150 mM NaCl. Germination was recorded daily. To assess the impact of salt stress on Soil Plant Analysis Development chlorophyll meter reading (SPAD) value, fresh weight, and Na^+^ content, one-week-old seedlings were treated with the same NaCl concentrations for 10 days. SPAD values were measured with a SPAD-502 Plus chlorophyll meter (Konica Minolta, Japan), and fresh weights were recorded. Plant growth was documented with photographs, and compound leaves were prepared to highlight specific details.

### 2.7 Determination of sodium ion content

Following 10 days of salt stress treatment, leaves were harvested and dried at 60°C for 7 days. Approximately 0.25 g of homogenized dried leaf tissue was accurately weighed and transferred into a 100 mL digestion tube. Subsequently, 5 mL of sulfuric acid (H_2_SO_4_) and hydrogen peroxide (H_2_O_2_) were added to the digestion tube. The tube was then placed in a digestion furnace, and the digestion was heated until the solution turned colorless or transparent. After removing the digestion tube from the furnace and allowing it to cool to room temperature, the digested solution was transferred to a 50 mL centrifuge tube. The solution volume was then adjusted to 50 mL with ultrapure water (i.e., volume make-up). Finally, the Na^+^ content was analyzed using an inductively coupled plasma optical emission spectrometer (ICP-OES, USA).

### 2.8 Expressions of salt resistance-related genes

To identify potential downstream genes associated with salt tolerance, including *GmSOS1*, *GmSALT3*, *GmHKT1*(High-affinity K^+^ Transporter 1), *GmAKT1*(*Arabidopsis* K^+^ Transporter 1), *GmNHX1*(Na^+^/H^+^ eXchanger family), and *GmNHX5*, qRT-PCR analysis was conducted on both the OE lines and WT plants subjected to 150 mM NaCl stress, based on previously established methodologies (Zhang et al., 2022; Liu et al., 2016; Chen et al., 2011; Feng et al., 2025; Sun et al., 2019, 2021). One-week-old seedlings were exposed to 150 mM NaCl treatment for varying durations of 12, 24, and 48 hours to capture dynamic transcriptional changes. Total RNA was extracted at each time point, reverse-transcribed into complementary DNA (cDNA), and subsequently used for qRT-PCR amplification using gene-specific primers (**Supplementary Table S2**). The relative expression levels of the target genes were normalized against internal reference genes, and the fold changes were calculated to assess transcriptional responses under salt stress over time.

### 2.9 Statistical analysis

Randomized block design was used in the experiment, and all experiments were repeated at least three times independently. The data are presented as the mean ± SD of three independent experiments. Statistical analysis was performed using one-way ANOVA and Student’s *t*-test (*p* < 0.05) by GraphPad Prism 6 (Boston, USA) and SPSS Statistics Ver. 22.0 (IBM, New York City, USA).

## 3 Results

### 3.1 Identification and bioinformatics analysis of the *GmNAC018*

**Figure 1:**
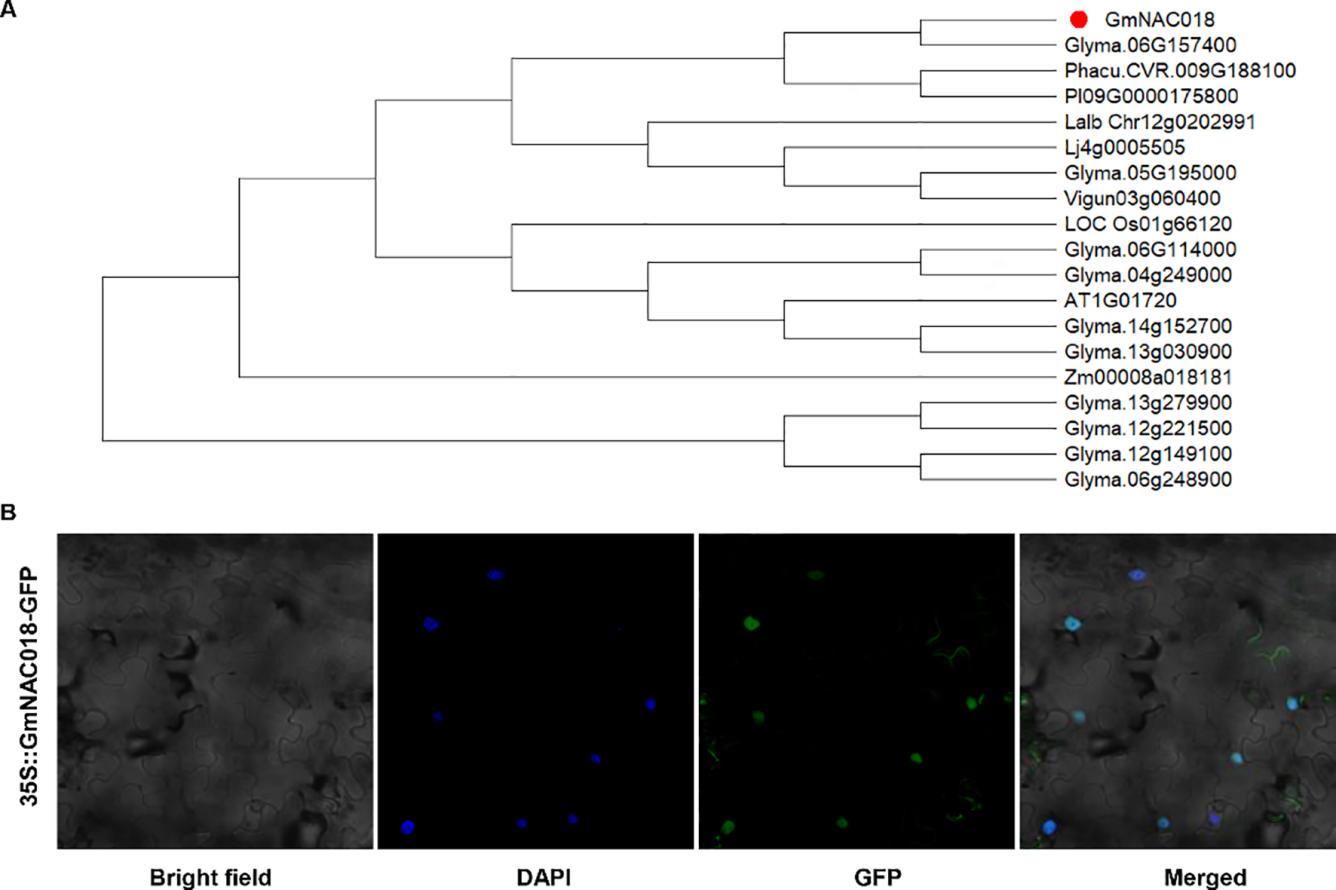
Bioinformatics analysis of the *GmNAC018*. **(A)** Phylogenetic tree of GmNAC018 and NAC members in other species. **(B)** Subcellular localization of GmNAC018. DAPI, as a nuclear dye, indicated the position of the nucleus. The specific vector was transformed into *N. benthamiana* leaf by an infiltration method.

Sequence analysis identified that *GmNAC018* comprises three exons and two introns, encoding a 292-amino acid peptide (**Supplementary Table S1**). The protein contains a NAM domain, spanning from amino acids 8 to 131, and has a predicted molecular weight of 33.65 kDa (**Supplementary Table S1**). The isoelectric point of the predicted GmNAC018 protein is 6.41, and its Grand Average of Hydropathy (GRAVY) is -0.758, indicating that GmNAC018 is a hydrophilic acidic protein. Structural model analysis revealed that the protein contains six distinct protein folds (**Supplementary Figure S1**). Phylogenetic analysis using NAC homologs from *Arabidopsis thaliana*, *O. sativa*, *Z. mays*, *G. max*, and other species showed that *GmNAC018* clusters closely with *Glyma.06G157400* and groups within the same clade as *AT1G01720*, confirming its classification into the SNAC-A (ATAF) subfamily (**Figure 1A**).

### 3.2 Subcellular localization of *GmNAC018*

The subcellular localization of the GmNAC018 protein was investigated by cloning the coding sequence (CDS) of *GmNAC018* from the soybean cultivar Huachun 6. A GmNAC018-GFP fusion construct, lacking the termination codon, was generated in the pCMBIA1302 vector and introduced into *N. benthamiana* leaves. Fluorescence analysis demonstrated that the GmNAC018-GFP fusion protein was localized in the nucleus (**Figure 1B**). These findings confirm that the GmNAC018 protein is localized to the nucleus, consistent with predictions by Plant-mPLoc.

### 3.3 Analysis of the expression pattern of *GmNAC018*

**Figure 2:**
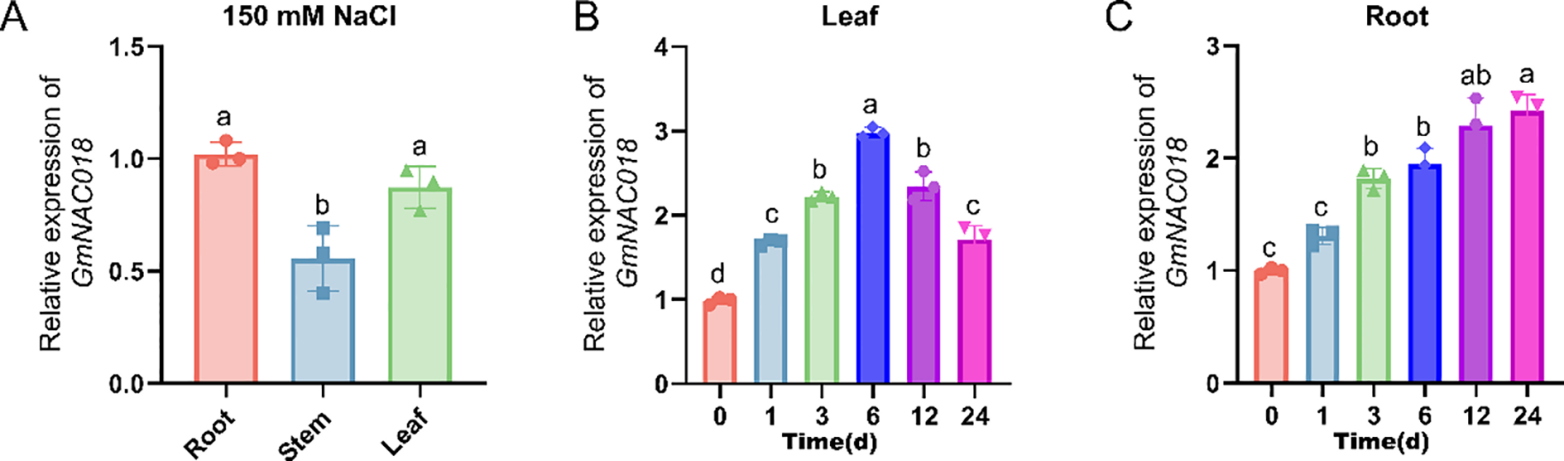
NaCl induces the expression of *GmNAC018* in soybean leaves and roots. **(A)** Tissue-specific expression of *GmNAC01*8 in roots, stems, and leaves at the V1 growth stage. **(B, C)** Time-course expression of *GmNAC018* in leaves **(B)** and roots **(C)** following treatment with 150 mM NaCl. The plant materials were collected after 0, 1, 3, 6, 12, and 24 hours under 150 mM NaCl treatment. Three independent biological replicates were performed, and the data are presented as means ± standard error (SE). One-way analysis of variance (ANOVA) and Student’s *t*-test (*p* < 0.05) were conducted. Different letters in the column indicate significant differences among the treatments at *p* < 0.05.

The qRT-PCR was conducted to evaluate the transcriptional levels of *GmNAC018* in Huachun 6 soybean plants. The analysis revealed that *GmNAC018* was predominantly expressed in both the roots and leaves (**Figure 2**). In the salt stress experiment, soybean plants were treated with 150 mM NaCl, and the expression of *GmNAC018* was monitored in the roots and leaves at multiple time points. The results showed that, in response to salt treatment, the mRNA levels of *GmNAC018* in the roots were rapidly induced, gradually increasing and peaking at 6 hours, followed by a decline. In contrast, in leaves, *GmNAC018* expression showed a steady increase over the 24 h period (**Figure 2**). These results suggest that the variations in *GmNAC018* expression in the leaves may be primarily influenced by salt uptake in the roots.

### 3.4 Determination of plant physiological parameters and phenotypes

**Figure 3:**
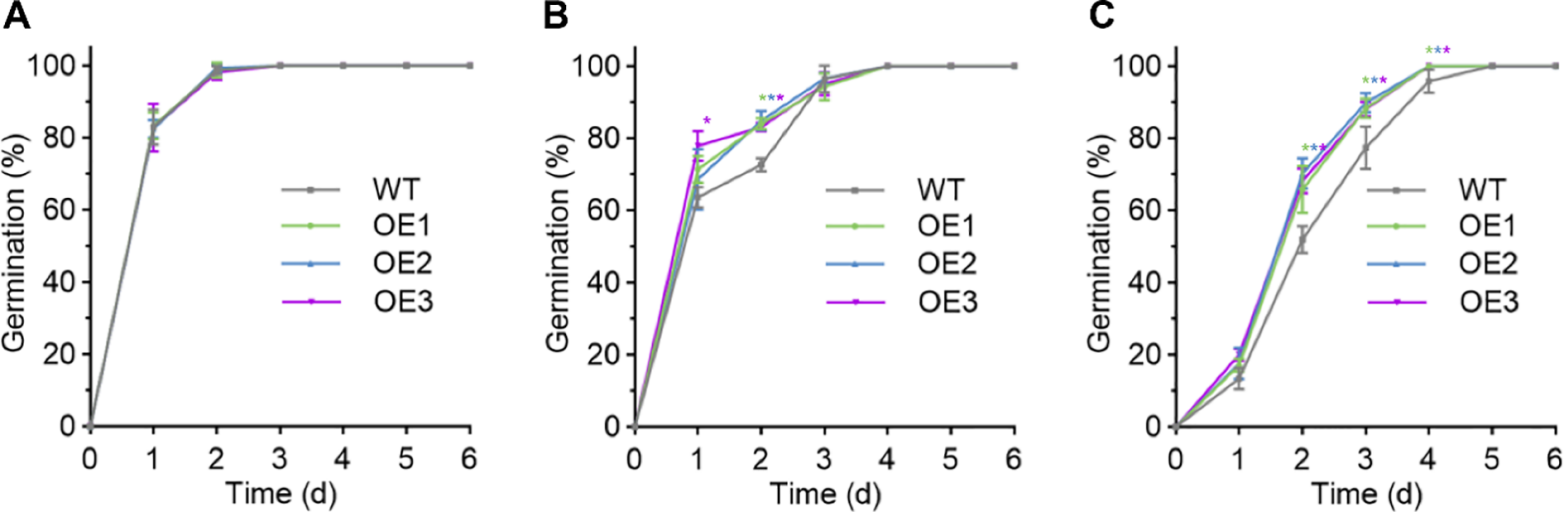
*GmNAC018* regulates soybean seed germination under salt stress. **(A-C)** Germination rates of WT and GmNAC018 overexpression lines (OE) over six days without **(A)**, or with 100 mM **(B)** and 150 mM **(C)** NaCl treatment. Three independent biological replicates were performed, and the data are presented as means ± standard error (SE). One-way analysis of variance (ANOVA) and Student’s *t*-test (*p* < 0.05) were conducted. Asterisks of different colors in the columns indicate significant differences between the OE lines and the WT (*p* < 0.05).

The function of *GmNAC018* in plant salt stress responses was investigated using ten *GmNAC018* OE soybean lines, which were selected through herbicide identification, PCR, and qRT-PCR. Three T_3_ generation OE lines (OE1, OE2, and OE3) with high expression levels were chosen for salt tolerance phenotype analysis. No significant difference in seed germination rates was observed between WT and OE lines in conventional vermiculite (**Figure 3A**). However, under 100 and 150 mM NaCl treatments, OE lines demonstrated higher germination rates than WT (**Figures 3B, C**). There were significant differences in seed germination rate between WT and OE lines at 2, 3, and 6 days under the treatment of 150mM NaCl (**Figure 3C**). Compared with the 150 mM NaCl treatment (**Figure 3C**), the difference in seed germination rates between the WT and OE was smaller under the 100 mM NaCl treatment (**Figure 3B**). Moreover, increased salt concentration further enhanced the salt tolerance observed in the *GmNAC018* OE lines.

**Figure 4:**
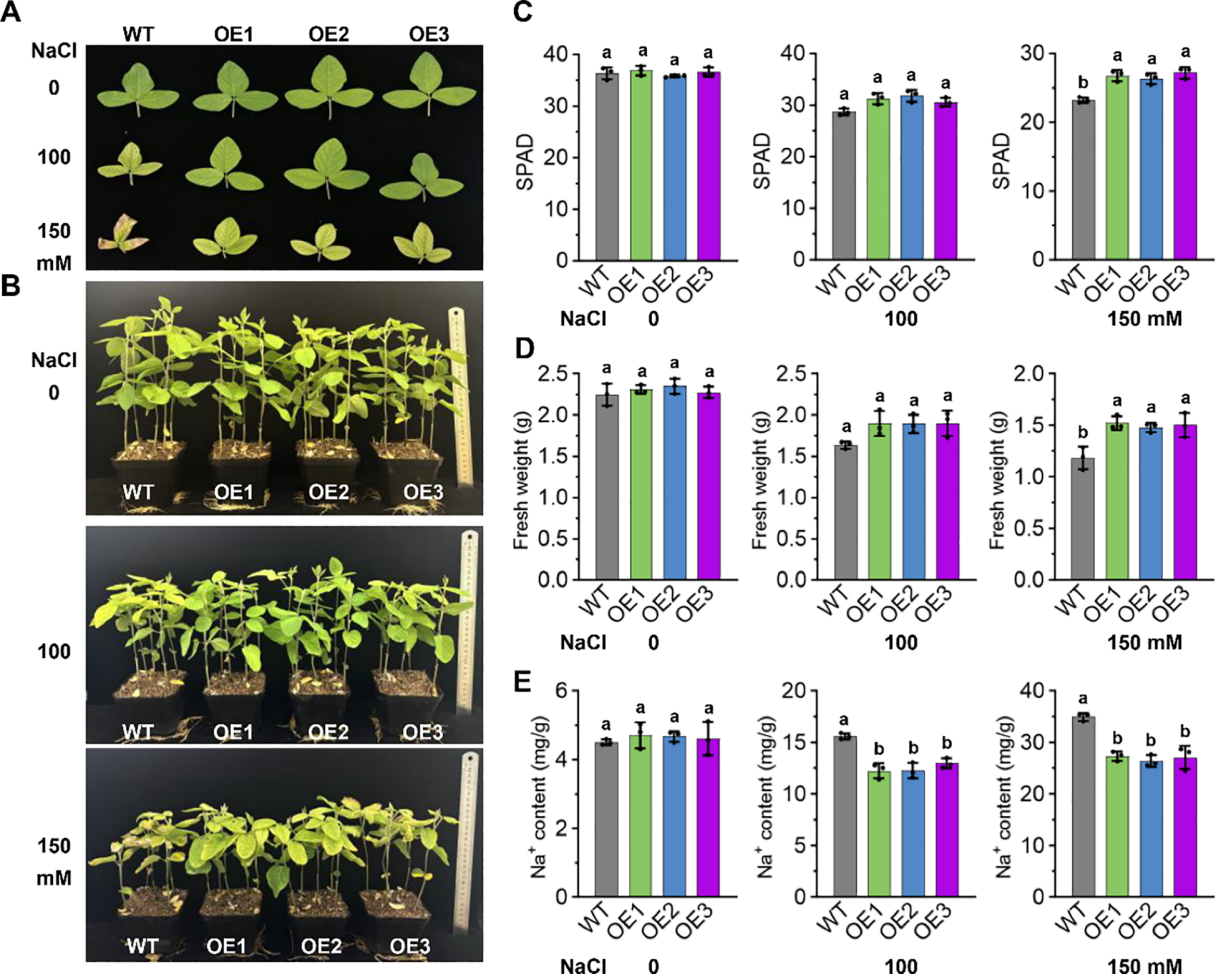
*GmNAC018* regulates the growth of soybean plants under salt stress. **(A)** Compound leaf phenotype. **(B)** Seedling phenotype. **(C)** Analysis of SPAD. **(D)** Analysis of fresh weight. **(E)** Analysis of Na^+^ content. The plant materials were collected after ten days under 0, 100 and 150 mM NaCl treatment. Three independent biological replicates were performed, and the data are presented as means ± standard error (SE). One-way analysis of variance (ANOVA) and Student’s *t*-test (*p* < 0.05) were conducted. Different letters in the column indicate significant differences among the treatments at *p* < 0.05.

To improve the narrative flow, consider adding a transitional sentence. For example: “Given that salt stress induces GmNAC018 expression, we next investigated its whether GmNAC018 is required for soybean growth under salt stress conditions. Two-week-old seedlings of the OE lines and WT were exposed to salt stress at concentrations of 0, 100, and 150 mM NaCl for ten days. Notable symptoms, such as yellowing of the leaves and stunted growth, were observed under salt stress. While the growth of the WT plants was significantly impaired, the transgenic lines exhibited less pronounced growth inhibition (**Figures 4A, B**). Upon measurement, both the OE lines and the WT showed a reduction in SPAD values compared to the control group under salt stress (**Figure 4C**). However, at each salt concentration, the OE lines maintained higher SPAD values than the WT, suggesting better chlorophyll retention. Furthermore, the fresh weight and Na^+^ content were also assessed. The OE lines exhibited a greater fresh weight and lower Na^+^ content than the WT (**Figures 4D, E**). There is a significant difference in Na^+^ content between WT and OE lines under the treatment of 100 and 150 mM NaCl.

### 3.5 *GmNAC018* positively regulates the expression of salt-responsive genes

**Figure 5:**
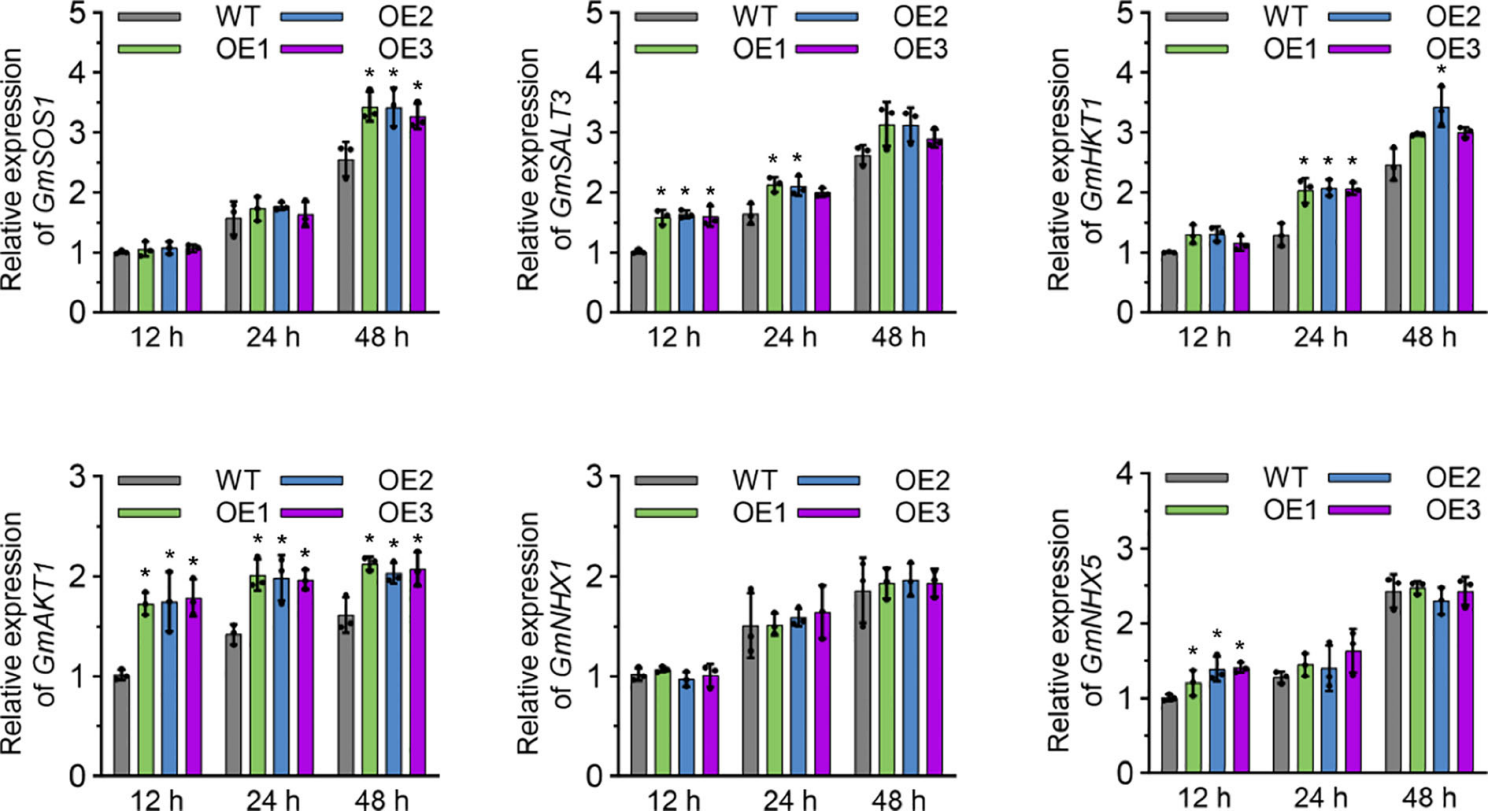
qRT-PCR analysis of the salt-responsive marker genes in soybean induced by salt stress. The plant materials were collected from one-week-old seedlings after 12, 24, and 48 hours under 150 mM NaCl treatment. Three independent biological replicates were performed, and the data are presented as means ± standard error (SE). One-way analysis of variance (ANOVA) and Student’s *t*-test (*p* < 0.05) were conducted. The asterisks in the column indicate significant differences among the OE lines and WT at *p* < 0.05.

To determine whether *GmNAC018* confers salt tolerance by regulating salt-related genes, we measured the expression levels of salt-responsive marker genes (*GmSOS1*, *GmSALT3*, *GmHKT1*, *GmAKT1*, *GmNHX1*, and *GmNHX5*) under salt stress. The results revealed that the expression levels of *GmAKT1* were significantly higher in OE lines than those in WT at 12, 24, and 48 h (**Figure 5**). While the expression levels of other genes, such as *GmSOS1*, *GmSALT3*, *GmHKT1*, and *GmNHX5*, were significantly different in each time period. It is worth noting that the expression level of *GmNHX1* between the OE lines and WT was not significant at three time points after treatment. These genes, whose expression patterns closely match those of *GmNAC018*, suggest that it may enhance salt tolerance by regulating downstream genes involved in salt stress.

## 4 Discussion

Soil salinization is a widespread and harmful abiotic stressor that significantly limits agricultural productivity worldwide (Singh, 2022). High salinity disrupts essential cellular functions by causing ion imbalance, osmotic stress, and the accumulation of reactive oxygen species. These effects result in reduced growth and considerable yield losses in crops such as soybean, which is of major economic importance (Faisal et al., 2024; El-Ramady et al., 2024). Plants have developed complex transcriptional regulatory networks to mitigate salt stress by controlling the expression of specific genes and transcription factors. The abscisic acid (ABA) signaling pathway, which includes transcription factors such as *ABFs*/*AREBs* and *ABI5*, is central to improving salt tolerance through the regulation of downstream gene expression (Collin et al., 2021). Additionally, the crosstalk between the ABA, JA, and ETH signaling pathways, mediated by key transcription factors like *MYC2* and *ERF1*, fine-tunes plant responses to salt stress (Kazan and Manners, 2013; Mao et al., 2016). Transcription factors from the NAC, MYB, and bZIP families coordinate the expression of ion transporters and osmoprotectant genes, supporting ion homeostasis and osmotic balance under saline conditions (Zheng et al., 2025; Jakoby et al., 2002). These regulatory networks collectively enable plants to adapt to high salinity environments and maintain productivity. Within these networks, transcription factors (TFs) serve as central orchestrators, governing the expression of myriad downstream effector genes that collectively mediate stress tolerance (Lindemose et al., 2013; Meshi and Iwabuchi, 1995). The NAC (NAM, ATAF1/2, CUC2) family of TFs, in particular, has been recognized for its important roles in plant development and responses to various abiotic stresses, including salinity (Rui et al., 2023; Shiriga et al., 2014; Sanjari et al., 2019; Nuruzzaman et al., 2010). The key NAC TFs like *SNAC-1* in rice, *TaNAC29* in wheat, and *ANAC019*, *ANAC055*, and *ANAC072* in *A. thaliana* have been demonstrated to significantly enhance salinity tolerance through the regulation of genes involved in ROS scavenging, ion homeostasis, osmotic adjustment, and stress signaling (Li et al., 2019; Huang et al., 2015; Jensen et al., 2010; Bu et al., 2008; Li et al., 2016). Genome-wide studies have identified NAC family members as contributors to oxidative stress responses under abiotic stress conditions (He et al., 2022; Ling et al., 2023). Some NAC genes have been isolated from soybean research, and transgenic plants show higher salt tolerance than wild-type plants at the seedling stage (Shi et al., 2025; Wang et al., 2022). However, limited research has addressed the response of soybean NAC family transcription factors to salt stress, and the underlying molecular mechanisms remain unclear.

In this study, the functional characteristics of soybean NAC transcription factor *GmNAC018* (*Glyma.04G208300*) were provided, and its regulatory role in enhancing salt tolerance was preliminarily clarified. As a member of the NAC family, *GmNAC018* shares 66.0% sequence similarity with *AtNAC2* and clusters phylogenetically within the SNAC-A/ATAF subgroup, consistent with its homology to *Glyma.06G157400* and *At1G01720* (Melo et al., 2018). In addition, in phylogenetic tree analysis, *GmNAC018* and an algae belong to one branch, which indicates that NAC family genes are highly conserved in different species, suggesting that they may have similar functions (**Figure 1**) (Fraga et al., 2021). Consistent with the prediction of nuclear location by online subcellular localization, the nuclear accumulation of GmNAC018-GFP fusion protein was confirmed by the coincidence of GFP fluorescence color and DAPI nuclear dye fluorescence in the experimental verification of transient expression in *N. benthamiana*. This result suggests its function as a transcription factor, which is consistent with the nuclear activity of SNAC-A homologues in stress signals (Nakashima et al., 2012). While it is similar to NAC family genes of other species in structure, such as conserved domains, it is speculated that *GmNAC018* may have a unique expression pattern and molecular function (**Figure 2**). Previous studies have reported root-specific expression of certain soybean NAC genes (Hao et al., 2011). However, our results substantiated that the expression of *GmNAC018* is not limited to roots, but also expressed in other tissues of plants, especially in leaves (**Figure 2A**), which is similar to some previous reports (Fraga et al., 2021). The expression of the NAC018 gene was rapidly induced in roots under 150 mM NaCl treatment, reached the peak at 6 hours under salt stress, and then the expression level in leaves continued to increase within 24 hours. This spatiotemporal pattern suggests that *GmNAC018* is activated early in root-based salt sensing and may subsequently regulate systemic responses in leaves.

To clarify the mechanism by which *GmNAC018* enhances plant salt tolerance, transgenic soybean lines were generated and subjected to salt stress treatment alongside wild-type controls. Experimental assessments included germination rate, phenotypic analysis, and physiological parameter measurements. Under salt stress, the germination rate of transgenic soybeans was inhibited but remained significantly higher than that of wild-type plants, particularly at high salt concentrations (**Figure 3**). Phenotypic analysis of plants showed that the OE line of *GmNAC018* showed significantly enhanced salt tolerance compared with wild plants. Under NaCl stress, the seedlings of over-expressed lines maintained a higher chlorophyll, thus maintaining green rather than yellow (**Figures 4A, B**). Salt stress has a significant effect on the chlorophyll content of plants, and SPAD value is a commonly used index to measure chlorophyll content (Shah et al., 2017). Typically, salt stress reduces chlorophyll content, leading to decreased photosynthetic efficiency and impaired plant growth and development (Taffouo et al., 2017; Hameed et al., 2021). The SPAD content of OE lines is higher than that of the WT, indicating that they are less damaged by salt stress (**Figure 4C**). It showed that the overexpression of *GmNAC018* enhances the tolerance of plants to salt stress. Physiological parameter analysis showed that under salt stress, the *GmNAC018* OE line accumulated more biomass and had lower Na^+^ content than wild-type plants. (**Figures 4D, E**). These phenotypic advantages suggest that *GmNAC018* reduces ion toxicity and growth inhibition. The findings indicate that *GmNAC018* may positively regulate salt tolerance in soybean by reducing Na^+^ absorption under salt stress. To further investigate whether *GmNAC018* confers salt tolerance through the regulation of salt-related genes, the expression levels of key salt-related genes were evaluated under salt stress. To further explore whether *GmNAC018* confers salt tolerance by regulating salt-related genes, we evaluated the expression levels of these genes under salt stress. The results showed that the expression levels of *GmSOS1*, *GmSALT3*, *GmHKT1*, *GmAKT1*, *GmNHX1*, and *GmNHX5* in over-expressed lines were significantly higher than those in wild-type plants, and this expression pattern was very similar to *GmNAC018* (**Figure 5**). The improved Na^+^ homeostasis in OE lines is associated with the synergistic upregulation of key ion transporters, and the time-dependent induction of these genes mirrors the early induction of *GmNAC018*, suggesting direct or indirect transcriptional regulation of these downstream effector factors.

We propose that *GmNAC018* acts as a master regulator in soybean salt adaptation by orchestrating the expression of genes critical for Na^+^ exclusion, Na^+^ transport, and vacuolar sequestration. This role is analogous to that of SNAC-A group homologs, such as *OsNAC6* in rice, which similarly enhance tolerance by modulating ion transport (Nakashima et al., 2007). The results of our overexpression experiment showed that *GmNAC018* overexpression was sufficient to enhance soybean salt tolerance. However, to finally confirm whether this tolerance mechanism is necessary under endogenous expression conditions, further functional deletion verification is still needed using the *GmNAC018* mutant. On this basis, the future work needs to confirm the biological function of salt tolerance of *GmNAC018* through experiments such as gene editing, verify the direct target of *GmNAC018* through experiments such as ChIP-seq, and explore its synergistic interaction with other stress pathways, so as to further clarify the complex regulatory network behind salt tolerance.

## 5 Conclusion

In this study, we identified the soybean NAC transcription factor gene *GmNAC018* and observed its significant up-regulation under salt stress. Overexpressing *GmNAC018* in transgenic soybean increased salt tolerance, likely by synergistically up-regulating key ion transporter genes. These findings suggest that *GmNAC018* acts as a central regulator in the salt stress response network and provide theoretical support for enhancing crop salt tolerance through molecular breeding.

## Data Availability

The original contributions presented in the study are included in the article/**Supplementary Material**. Further inquiries can be directed to the corresponding authors.
