# Supplementary material for: The transcription factor GmNAC018 confers salt tolerance in soybean
Source: Front Plant Sci. 2026 Jan 19;16:1728235. doi: 10.3389/fpls.2025.1728235 (PMC12863262; doi:10.3389/fpls.2025.1728235)
Supplement: Supplementary file 1 [file DataSheet1.docx]

**Figure S1. Prediction of the *GmNAC018* protein structure model.** One color represents one protein fold.


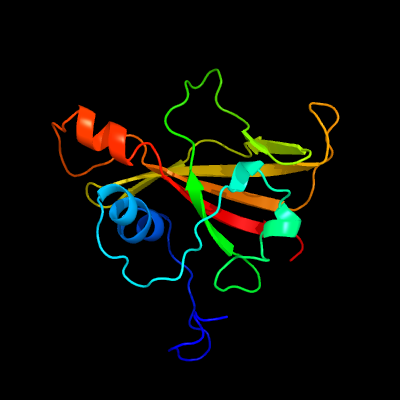


**Table S1. The sequencing information of *GmNAC018*.**

| **Genomic sequence：**  ATGAAGGGAGAATTAGAGTTGCCACCTGGGTTCAGATTTCACCCCACTGATGAAGAATTGGTGAATCACTACTTGTGTAGGAAGTGCGCTGGTCAACCAATCGCGGTTCCCGTCATCAAAGAGGTCGATTTGTACAAGTTTGATCCATGGCAGCTTCCAGGTTCGTTTAGTTTCTTATGGGTTTCGTTTTGTGTGAAACCAATTCCAATTGTATCGTTTAGTTCAATTGATTGATTATGAGTTGGTCAATGCAGAAATTGGTTTTTACGGCGAGAAAGAATGGTACTTCTTTTCTCCTCGGGACCGGAAATACCCGAACGGTTCACGGCCGAACCGCGCCGCCGGAAGCGGCTATTGGAAAGCCACCGGCGCTGATAAACCGATCGGAAAACCGAAAGCGCTTGGGATCAAGAAAGCTCTGGTTTTTTACGCCGGAAAAGCCCCCAAAGGTGTGAAAACCAATTGGATTATGCACGAATATCGCCTCGCCAATGTTGACCGATCTGCCTCCAAGAAAAAAAACAACAACTTGAGGGTATGTCCCAAATTGGAATTTCACTAAAATCTAGATTAGTTTGCTTAAGTGTTTTTTAATCAACCAAGCGGGTCTAGCATAAAAAGTTTTCGTTTTTAATCACAATTAGAGTTTCACTTATATAACATATGCAATTTTAATTGTTTCCCAATCAAAATTAGAATTGCATAAGTCTTGTTCTATAATTTTTATAAAGAAGGATCTGATTTTGTTGTGTTATGGTGCACAGCTTGATGATTGGGTGTTGTGTCGAATCTACAACAAGAAAGGGAAGATTGAGAAATACAACACAGGCGCAGCGAAGATGAATGTTGAGATGGTTCATAGTTTTGAGCACGAGAACGAGACGAAGCCAGAGATTCATAAGCTAGGAAATGAGCAATTGTACATGGAGACTTCGGATTCGGTGCCAAGGTTGAACACGGACTCGAGCAGTTCGGAGCACGTGGTTTCGCCCGATGTCACGTGCGAGAGGGAGGTGCAGAGCGACCCCAAGTGGAACGATGATCTGGACCTAAAGCTAGAAAACGCGTTTGATTTTCAGTTTAATTACTTGGACGATAATAACCTTTCCGTGGATGATTACCTTTTTGGCACTGTTCAGTATCAAATGGGCCAGCTCTCGCCCTTGCAGGACATGTTCATGTACCTACAGAAGATGTGA  **Note: Introns are marked in blue and exons are marked in black.**  **CDS sequence：**  ATGAAGGGAGAATTAGAGTTGCCACCTGGGTTCAGATTTCACCCCACTGATGAAGAATTGGTGAATCACTACTTGTGTAGGAAGTGCGCTGGTCAACCAATCGCGGTTCCCGTCATCAAAGAGGTCGATTTGTACAAGTTTGATCCATGGCAGCTTCCAGAAATTGGTTTTTACGGCGAGAAAGAATGGTACTTCTTTTCTCCTCGGGACCGGAAATACCCGAACGGTTCACGGCCGAACCGCGCCGCCGGAAGCGGCTATTGGAAAGCCACCGGCGCTGATAAACCGATCGGAAAACCGAAAGCGCTTGGGATCAAGAAAGCTCTGGTTTTTTACGCCGGAAAAGCCCCCAAAGGTGTGAAAACCAATTGGATTATGCACGAATATCGCCTCGCCAATGTTGACCGATCTGCCTCCAAGAAAAAAAACAACAACTTGAGGCTTGATGATTGGGTGTTGTGTCGAATCTACAACAAGAAAGGGAAGATTGAGAAATACAACACAGGCGCAGCGAAGATGAATGTTGAGATGGTTCATAGTTTTGAGCACGAGAACGAGACGAAGCCAGAGATTCATAAGCTAGGAAATGAGCAATTGTACATGGAGACTTCGGATTCGGTGCCAAGGTTGAACACGGACTCGAGCAGTTCGGAGCACGTGGTTTCGCCCGATGTCACGTGCGAGAGGGAGGTGCAGAGCGACCCCAAGTGGAACGATGATCTGGACCTAAAGCTAGAAAACGCGTTTGATTTTCAGTTTAATTACTTGGACGATAATAACCTTTCCGTGGATGATTACCTTTTTGGCACTGTTCAGTATCAAATGGGCCAGCTCTCGCCCTTGCAGGACATGTTCATGTACCTACAGAAGATGTGA  **Peptide sequence：**  MKGELELPPGFRFHPTDEELVNHYLCRKCAGQPIAVPVIKEVDLYKFDPWQLPEIGFYGEKEWYFFSPRDRKYPNGSRPNRAAGSGYWKATGADKPIGKPKALGIKKALVFYAGKAPKGVKTNWIMHEYRLANVDRSASKKKNNNLRLDDWVLCRIYNKKGKIEKYNTGAAKMNVEMVHSFEHENETKPEIHKLGNEQLYMETSDSVPRLNTDSSSSEHVVSPDVTCEREVQSDPKWNDDLDLKLENAFDFQFNYLDDNNLSVDDYLFGTVQYQMGQLSPLQDMFMYLQKM*  **Note: The blue letters indicate the NAM domain (amino acids 8-131).** |
| --- |

**Table S2. Primers used in the study.**

| Primer Name | Primer Sequence (5′ to 3′) | Purpose |
| --- | --- | --- |
| pTF101-GmNAC018-F | gagaacacgggggactctagaATGAAGGGAGAATTAGAGTTGCCA | Cloning |
| pTF101-GmNAC018-R | cgatcggggaaattcgagctcTCACATCTTCTGTAGGTACATGAACATG |  |
| pCAMBIA1302GmNAC018-F | acgggggactcttgaccatggATGAAGGGAGAATTAGAGTTGCCA | Subcellular localization |
| pCAMBIA1302GmNAC018-R | aagttcttctcctttactagtCCATCTTCTGTAGGTACATGAACATG |  |
| qGmNAC018F | GAAGGCTACCGGAGCTGAC | qRT-PCR |
| qGmNAC018R | CTTCACTCCCTTTGGGGCT |  |
| Acting-Gm-F | GCACCACCGGAGAGAAAATA |  |
| Acting-Gm-R | GTGCACAATTGATGGACCAG |  |
| qGmSOS1F | CTGCTTACTGGGAGATGCTTGA |  |
| qGmSOS1R | TTCGGTGGGAACATACTGGAC |  |
| qGmSALT3F | CGTGTTGCTCGCAAGTGTTCT |  |
| qGmSALT3R | TCCTCAATCCTCCTCGTCATTCTATT |  |
| qGmAKT1F | ATGTTGGTGTCCATGTCCGTGTG |  |
| qGmAKT1R | TGGCTCCAAGGGAAGGTAGTATT |  |
| qGmHKT1F | TTCATCCCCACCAACGAGA |  |
| qGmHKT1R | GTGACCCTTTTCAGAGCCATTA |  |
| qGmNHX1F | ACCTCAGACCATGCCTCCG |  |
| qGmNHX1R | TCATCCACCGATTCTCCTCA |  |
| qGmNHX5F | GTCTGGGTTCAGTCTCGCAC |  |
| qGmNHX5R | ATCAGAAAGAGCAAGCCACCA |  |
| PTF101-F | CCACCGGCGCTGATAAACCG | Molecular identification |
| PTF101-R | CAACACCCAATCATCAAGCCTC |  |
| p1302-F | GGTCGATTTGTACAAGTTTG |  |
| p1302-R | TGTTCAACCTTGGCACCG |  |
